# Posterior condylar cartilage thickness determines accuracy of femoral component rotation during total knee arthroplasty in valgus knees: Comparison with varus knees

**DOI:** 10.1002/jeo2.70486

**Published:** 2025-11-03

**Authors:** Teruyuki Miyasaka, Taiki Neyatani, Toshiyuki Omori, Tomohiro Kayama, Daisuke Kubota, Mitsuru Saito

**Affiliations:** ^1^ Department of Orthopaedic Surgery Jikei University School of Medicine Tokyo Japan

**Keywords:** cartilage thickness, femoral component rotation, kinematic alignment, posterior condylar cartilage, total knee arthroplasty

## Abstract

**Purpose:**

Accurate femoral component (FC) rotation is critical in total knee arthroplasty (TKA). Residual posterior condylar cartilage is difficult to assess intraoperatively and may bias the posterior condylar axis. Quantitative data remain scarce, particularly for lateral compartment osteoarthritis, and the assumption of a uniform 2‐mm cartilage thickness has rarely been tested.

**Methods:**

Twenty‐five valgus knees in 22 Asian women undergoing primary TKA were analysed; 31 previously measured female varus knees, assessed with the same protocol, served as controls. After posterior femoral resection, resected condyles were radiographed perpendicular to the cut. Medial (*m*) and lateral (*l*) cartilage thicknesses were measured with magnification adjustment. The thickness difference (*C*_diff_) was regressed against theoretical rotational deviation (*θ*). The slope and intercept were compared between valgus and varus cohorts. The proportion of knees within a prespecified ‘2 mm range’ (1.5–2.5 mm) was calculated.

**Results:**

FC rotation shifted 1.31° per 1‐mm cartilage asymmetry (95% confidence interval [CI]: 1.28–1.35; *R* ^2^ = 0.99). Slopes were similar in varus and valgus cohorts. The mean *θ* was 1.6° ± 1.3°, with values up to 4°. Thin cartilage (<1.5 mm) was more frequent in varus than valgus (42% vs. 24%), not significant. Overall, ≈52% of knees fell within the ‘2 mm range’ (1.5–2.5 mm).

**Conclusion:**

Residual posterior condylar cartilage directly affects FC rotation in TKA. In Asian women, each 1‐mm medial–lateral asymmetry produced ≈1.3° of rotational error irrespective of deformity pattern. Only half of the knees approximated 2 mm, indicating that a fixed 2‐mm correction is not universally applicable.

**Level of Evidence:**

Level III.

**Abbreviations**
CI: confidence interval
CT: computed tomography
FC: femoral component
HKA: hip–knee–ankle
ICC: intraclass correlation coefficient
MRI: magnetic resonance imaging
OA: osteoarthritis
PCA: posterior condylar axis
TKA: total knee arthroplasty

## INTRODUCTION

Accurate rotational alignment of the femoral component (FC) is crucial in total knee arthroplasty (TKA), given its considerable influence on patellar tracking, flexion gap balancing and overall knee kinematics [1, 2, 4, 12, 17]. The transepicondylar axis and Whiteside's line are widely used as anatomical landmarks but are difficult to identify intraoperatively. Therefore, most TKA systems rely on the posterior condylar axis (PCA) during surgery for determining FC rotational alignment. However, the thickness of the residual cartilage on the posterior condyles may vary between the medial and lateral sides, potentially affecting the measured PCA and causing deviations from preoperative planning. Several studies have assessed cartilage morphology using imaging modalities such as computed tomography (CT) arthrography and magnetic resonance imaging (MRI). Others have used trigonometric methods to quantify the angular impact, directly measuring residual cartilage thickness on resected posterior condyles after bone cuts by incising the remnants with a scalpel [3, 8, 11, 22].

In a previous investigation of varus knees [18], the distance between the posterior condylar tips was measured, along with medial cartilage thickness (*m*) and lateral cartilage thickness (*l*). Trigonometric calculations revealed that the intercondylar distance (*d*) influenced cartilage thickness and affected FC rotational positioning. However, there have been no quantitative investigations of this problem in lateral compartment osteoarthritis (OA). Although Nam et al. [20] reported the influence of posterior condylar cartilage thickness on rotational alignment of the FC, the minimal cartilage thickness observed in varus knees conflicted with previous reports [3, 8, 11, 22].

In an earlier study by another group, Nam et al. [19] reported that the thickness of unworn cartilage in the posterior femoral condyle was consistently around 2.0 mm, regardless of coronal knee alignment. In contrast, Klasan et al. [13] showed frequent deviation from the 2‐mm reference, and a recent prospective intraoperative study by Campi et al. [5] reported a mean thickness of 2.6 ± 0.7 mm (range: 1.5–5.0 mm) in the unworn compartment, thereby challenging the idea of a uniform 2‐mm correction.

To extend previous findings to valgus knees, a radiographic trigonometry‐based method [8] was applied to measure posterior condylar cartilage thickness in lateral compartment OA, and its effect on FC rotation was examined, using medial compartment OA as a reference [18].

Despite these prior imaging and intraoperative studies, the size‐adjusted mapping between the signed medial–lateral residual cartilage difference (*C*_diff_) and theoretical FC rotational deviation (*θ*) in TKA—and whether this relation varies by deformity pattern (valgus vs. varus)—has not been quantified. Estimates of how often knees with cartilage thickness that fall within the ‘2 mm range’ (1.5–2.5 mm) remain limited. Because preoperative planning and intraoperative workflows often adopt a uniform 2‐mm correction, its validity depends on this prevalence; if the range captures only a modest share of knees, uniform correction could introduce systematic bias, particularly in small‐statured populations. Here, using a single‐exposure radiographic method in a female‐only cohort encompassing valgus and varus knees, the slope was quantified relating *C*_diff_ to *θ*, its consistency across deformity patterns was tested, and the actual prevalence of knees within the ‘2 mm range’ was estimated.

It was hypothesised that (1) residual posterior condylar cartilage thickness differs systematically between varus and valgus knees with a measurable influence on FC rotational alignment and (2) the commonly used 2‐mm correction is not representative in small‐statured populations.

## MATERIALS AND METHODS

### Study design

The study protocol was approved by the Institutional Review Board of The Jikei University School of Medicine (Approval No. 33‐464 [11091]). Written informed consent was obtained from all participants. The study included 25 knees in 22 female Asian patients who underwent primary TKA for lateral compartment OA at The Jikei University School of Medicine between April 2022 and March 2025. Patients with a history of periarticular fractures or previous osteotomy around the knee were excluded.

Data for 31 knees in female patients with medial compartment knee OA from a previously collected data set served as a control group. These patients had been evaluated using the same radiographic methods and measurement protocols as the current valgus group. Thus, this study could specifically compare female patients with lateral and medial knee OA. Basic demographic information for both groups is given in Table 1 (see Results).

**Table 1:** Demographic characteristics of female patients with medial and lateral compartment knee osteoarthritis.

Group	*N*	Age (years)	Height (cm)	Weight (kg)	BMI (kg/m^2^)	HKA angle (°)	|HKA angle – 180| (°)
Medial OA	31	76.7 ± 6.3	148.9 ± 5.7	58.4 ± 9.0	26.3 ± 3.8	191.5 ± 6.4	11.6 ± 6.3
		(57, 85)	(133, 164)	(37, 82)	(21, 37)	(179, 206)	(1, 26)
Lateral OA	25	72.7 ± 8.0	153.7 ± 6.7	57.5 ± 11.6	24.3 ± 4.0	166.8 ± 8.3	13.2 ± 8.3
		(56, 83)	(142, 167)	(38, 78)	(16, 31)	(149, 179)	(1, 31)
*p* value		<0.05*	<0.01**	0.76	0.053	<0.001***	0.44

*Note*: Values are reported as the mean ± standard deviation (range).

Abbreviations: BMI, body mass index; HKA angle, hip–knee–ankle angle; OA, osteoarthritis.

* *p* < 0.05

** *p* < 0.01

*** *p* < 0.001.

### Sample size calculation and power analysis

Effect‐size (SESOI) and variance assumptions. *Δθ* = 1.5° was prespecified as the smallest effect size of interest, supported by previously published rotational offsets (~1.1°–1.7°) [3, 8, 11, 22]. For conservative planning, an SD = 1.7° was assumed, corresponding to Cohen's *d* = 0.88 and a required *n* = 21 knees per group (two‐sample, two‐sided *α* = 0.05, 1 − *β* = 0.80).

### Data collection

**Figure 1:**
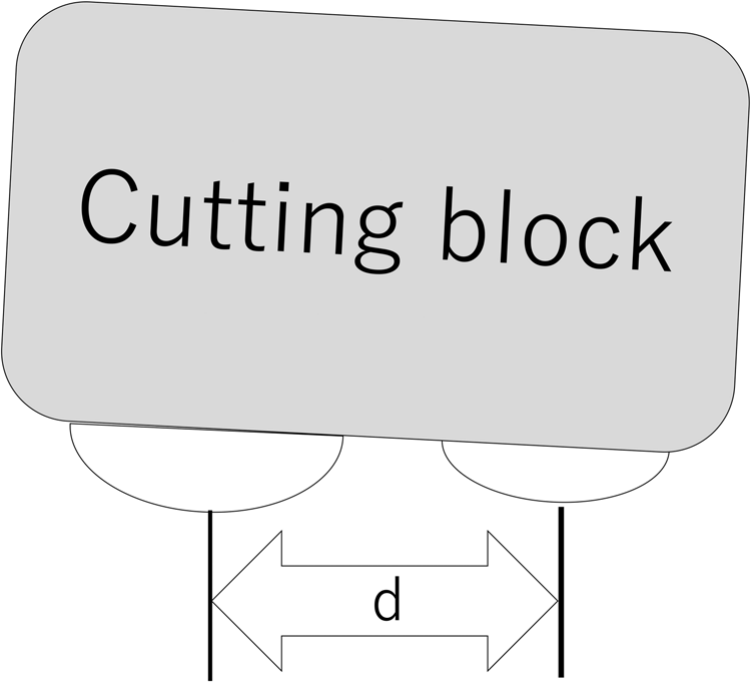
Measurement of the posterior femoral intercondylar distance (*d*).

Routine TKA was performed. The distal femur was resected using an intramedullary alignment rod, followed by proximal tibial osteotomy with an extramedullary alignment guide. Intraoperatively, after achieving an adequate extension gap, the posterior femoral intercondylar distance (*d*) was measured before the posterior condylar cuts. The tips of the Medial and lateral condyles were marked, and the distance between the tips was defined as *d* (Figure 1). *d* was measured once before posterior cuts to avoid bias from repeated measurements; before recording, the primary and assistant surgeons jointly verified correct placement of a rigid ruler on the most posterior points of the medial and lateral femoral condyles. The posterior femoral condyles were subsequently resected using an alignment guide referencing the PCA.

**Figure 2:**
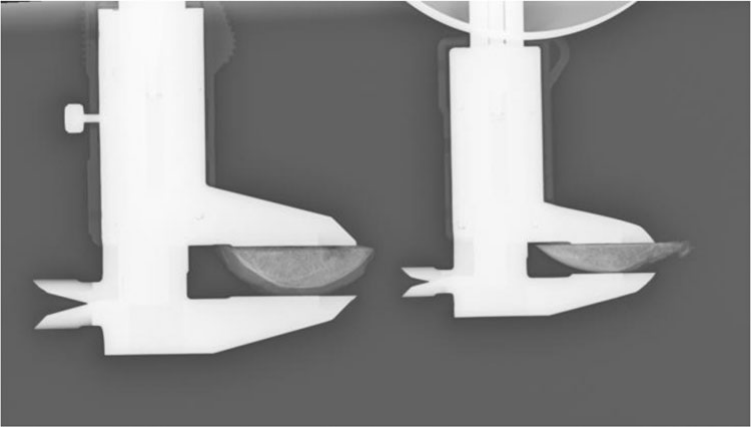
Radiographic setup for measurement of cartilage thickness.

**Figure 3:**
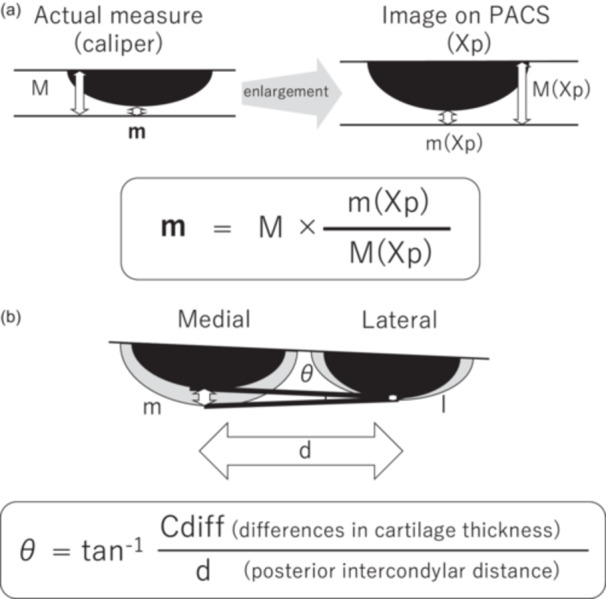
(a) Schematic representation of the method used for measuring cartilage thickness and correcting magnification on radiographic images. *M* = actual thickness of bone plus cartilage; *m* = actual cartilage thickness; *M* (*Xp*) = measured thickness of bone plus cartilage on radiograph (PACS); *m* (*Xp*) = measured cartilage thickness on radiograph (PACS). (b) Schematic representation of the effect of residual cartilage thickness on rotational alignment of the femoral component, illustrating the theoretical rotational deviation (*θ*). *C*_diff_ was defined as the cartilage thickness difference between medial and lateral condyles (*C*_diff_ = *m* − *l*). PACS, picture archiving and communication system.

Postoperatively, each sample was placed on a stable table with its surface parallel to the ground. The portion with the greatest cartilage thickness was secured using a digital caliper, and the orientation of the specimen was adjusted so that the cut surface was perpendicular to the caliper axis. A flat, stable Petri dish was then placed beneath the caliper, and the main scale was firmly positioned on the dish to ensure stable and perpendicular alignment with the film. The sample was positioned directly under the X‐ray beam, and irradiation was performed perpendicular to the ground. After imaging, the cut surface was confirmed to be in a straight line, indicating true perpendicularity (Figure 2). Although the radiographic images were magnified, the ratio of cartilage to bone thickness remained constant, allowing accurate measurements of thickness (Figure 3a).

The theoretical rotational deviation (*θ*) resulting from the difference in residual cartilage thickness between the medial and lateral posterior femoral condyles (*C*_diff_) was calculated using the trigonometric formula described by Fujii et al. [8] (Figure 3b).

The valgus knee cohort comprised 25 knees enroled at The Jikei University School of Medicine (Tokyo, Japan). For comparison, 31 female varus knees were extracted from a previously published data set [18] at Tokyo Metropolitan Toshima Hospital (Tokyo, Japan), a related facility where the authors also performed the surgeries. Although data were collected from two institutions, the radiographic protocols, surgical indications and measurement procedures were identical, allowing a direct comparison.

Sexual dimorphism in femoral condylar morphology and cartilage thickness is well documented; women generally have thinner articular cartilage and a narrower intercondylar distance than men [6, 7, 18]. Therefore, including both sexes would have introduced a significant confounder and might erroneously attribute sex‐related differences to compartmental disease. Because the present valgus (lateral OA) cohort included only female patients, the female subset from an earlier varus (medial OA) study [18] was retrospectively extracted to obtain two sex‐matched cohorts. This approach eliminated sex as a confounding variable, reduced biological variability and allowed any observed differences in cartilage thickness to be ascribed more confidently to compartment‐specific pathology.

Two board‐certified orthopaedic surgeons (A and B) measured each image twice in separate, blinded sessions; the mean of the four readings served as the reference value.

### Statistical analysis

Data distribution was assessed using the Shapiro–Wilk test. For consistency with reporting standards in this field, all continuous variables are expressed as mean ± standard deviation (SD), regardless of normality.

Intra‐ and interobserver reproducibility were assessed using the intraclass correlation coefficient (ICC) with a two‐way random‐effects model for absolute agreement [ICC (2, 1)]. ICCs were interpreted as poor (< 0.50), moderate (0.50–0.75), good (0.75–0.90) or excellent (>0.90) [14].

Linear regression analysis was used to evaluate the relationship between the medial–lateral cartilage thickness difference (*C*_diff_, defined as medial minus lateral) and *θ*, with regression slope, 95% confidence interval (CI) and *R* ^2^ values reported. Subgroup analyses were performed separately for varus and valgus knees to assess the consistency of the regression slope.

Continuous variables, including cartilage thickness (*m*, *l*), *C*_diff_, *d* and *θ* were compared between the medial and lateral compartment OA groups using Welch's *t*‐test. All statistical analyses were performed with JMP® version 18.2.1 (SAS Institute Inc.). Two‐sided *p* value of < 0.05 was considered statistically significant.

## RESULTS

### Demographic data

The mean age, height, body mass index (BMI) and preoperative hip–knee–ankle (HKA) angle in the valgus cohort were 72.7 ± 8.0 years, 153.7 ± 6.7 cm, 24.3 ± 4.0 kg/m^2^ and 166.8° ± 8.3°, respectively. The absolute deviation from 180° ( | HKA angle – 180 | ) was 13.2° ± 8.3°. Demographic data for both valgus and varus cohorts are summarised in Table 1.

### Reliability of measurements

Intraobserver and interobserver reproducibility was excellent for all variables. ICCs [ICC (2, 1) > 0.95] and mean absolute differences in paired measurements for observers A and B are summarised in Supporting Information S1: Table S1.

### Cartilage thickness and intercondylar distance

The mean medial condylar cartilage thickness (*m*) was 1.8 ± 0.9 mm (range: 0.0 to 3.4) and the mean lateral condylar thickness (*l*) was 0.6 ± 0.7 mm (range: 0.0 to 2.8) in knees with lateral compartment OA. The mean difference in cartilage thickness between the medial and lateral sides (*C*_diff_) was 1.2 ± 1.0 mm (range: –0.3 to 3.3).

The mean *d* was 44.3 ± 2.7 mm (range: 39.0 to 50.0), and the average impact of cartilage remnants on femoral rotation was 1.6° ± 1.3° (range: –0.5 to 4.1). Statistically significant intergroup differences were observed for *m*, *l* and *C*_diff_, all *p* < 0.001.

However, there were no significant differences in the absolute value of the cartilage thickness difference (| *M* − *L* |), *d* or *θ* (Table 2).

**Table 2:** Comparison of cartilage thickness, posterior intercondylar distance and rotational deviation between medial and lateral compartment osteoarthritis groups including women only.

Group	*N*	*m* (mm)	*l* (mm)	*C*_diff_ (mm)	|*C*_diff_| (mm)	*d* (mm)	*θ* (°)
Medial OA	31	0.4 ± 0.4 (0.0, 1.7)	1.7 ± 0.6 (0.4, 3.0)	−1.3 ± 0.7(−2.8, −0.2)	1.3 ± 0.7 (0.2, 2.8)	44.2 ± 2.0 (39.0, 48.0)	1.7 ± 0.9 (0.3, 3.9)
Lateral OA	25	1.8 ± 0.9 (0.0, 3.4)	0.6 ± 0.7 (0.0, 2.8)	1.2 ± 1.0 (−0.3, 3.3)	1.3 ± 0.9 (0.0, 3.3)	44.3 ± 2.7 (39.0, 50.0)	1.6 ± 1.3 (−0.5, 4.1)
*p* value		<0.001***	<0.001***	<0.001***	0.98	0.85	0.79

*Note*: Values are presented as the mean ± standard deviation (range).

Abbreviations: *C*_diff_, medial minus lateral cartilage thickness difference; *d*, posterior intercondylar distance; *l*, lateral cartilage thickness; *m*, medial cartilage thickness; OA, osteoarthritis; *θ*, theoretical rotation deviation angle.

*** *p* < 0.05 indicates a statistically significant difference.

### Relationship between cartilage asymmetry and rotational deviation

**Figure 4:**
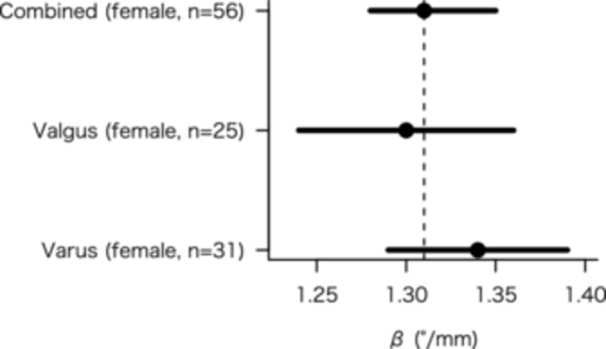
Forest plot showing regression slopes (*β*, °/mm) with 95% confidence intervals (CIs) for female subgroups (valgus vs. varus). The dashed line marks the combined‐female reference (*β* = 1.31°/mm). *β* denotes degrees of internal rotation per 1‐mm increase in *C*_diff_. *C*_diff_ = medial minus lateral cartilage thickness.

When the data for the varus and valgus groups were combined, a strong linear relationship was observed between *C*_diff_ and *θ*, with a slope of 1.31° per millimetre (*β* = 1.31, 95% confidence interval [CI]: 1.28–1.35; *p* < 0.001, *R* ^2^ = 0.99) (Figure 4). Subgroup analysis yielded similar slopes for both varus and valgus knees (1.34 vs. 1.30°/mm), indicating that the 1.3°‐per‐millimetre rule can be applied independent of coronal knee alignment (Table 3).

**Table 3:** Subgroup‐specific regression slopes (*β*), 95% confidence intervals (CIs), coefficients of determination (*R* ^2^) and *p* values for the association between medial–lateral cartilage‐thickness difference (*C*_diff_) and theoretical femoral‐component rotation (*θ*).

Group	*Β* (°/mm)	95% CI	*R* ^2^	*p* value
Combined (F, *n* = 56)	1.31	1.28–1.35	0.989	<0.001
Valgus (F, *n* = 25)	1.30	1.24–1.36	0.988	<0.001
Varus (F, *n* = 31)	1.34	1.29–1.39	0.991	<0.001

*Note*: *β* is expressed in degrees of internal rotation per 1‐mm increase in cartilage‐thickness asymmetry.

Abbreviations: *C*_diff_, medial minus lateral cartilage thickness difference; F, female.

### Comparison between varus and valgus knees

**Figure 5:**
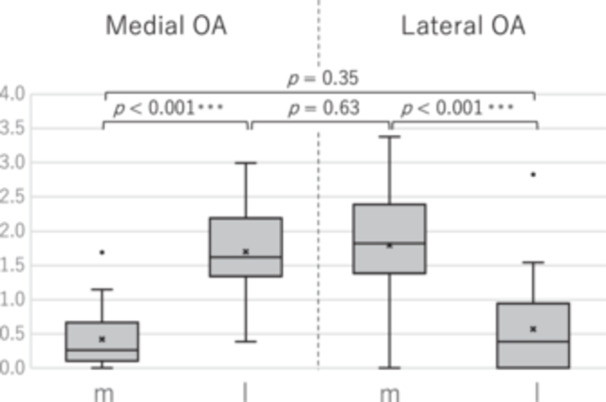
Comparison of residual cartilage thickness at the posterior condyles between medial and lateral OA groups including women only. OA, osteoarthritis.

There were no significant differences between the severely worn medial condyle in the medial‐compartment OA group and the severely worn lateral condyle in the lateral‐compartment OA group or between the mildly worn (relatively preserved) lateral condyle in the medial‐compartment OA group and the mildly worn medial condyle in the lateral‐compartment OA group (*p* = 0.35 and *p* = 0.63, respectively; Welch's *t*‐test) (Figure 5). For context, prior varus‐knee literature is summarised in Table 4, and the distribution in the female cohort of this study is shown in Table 5. By deformity, the distribution of residual posterior condylar cartilage categories (<1.5, 1.5–2.5, >2.5 mm) did not differ significantly (overall *χ* ^2^, *p* = 0.29; Table 5). Thin cartilage (<1.5 mm) was more frequent in varus than in valgus knees (42% [13/31] vs. 24% [6/25]; Fisher's exact *p* = 0.26), whereas cartilage > 2.5 mm was more common in valgus than in varus (20.0% [5/25] vs. 9.7% [3/31]; *p* = 0.45). The proportion of knees with residual posterior condylar cartilage that fell within the presumed ‘2 mm range’ was 56.0% (14/25; 95% CI: 37.1%–73.3%) in the valgus group and 48.4% (15/31; 95% CI: 32.0%–65.2%) in the varus group. Overall, only 51.8% (29/56; 95% CI: 39.0%–64.3%) of knees fell within this range. Exact binomial tests indicated that the widely assumed threshold of ≥ 70% was not met: the hypothesis of a ≥70% prevalence was statistically rejected for the varus group (*p* = 0.0095) and for the cohort as a whole (*p* = 0.0031), while the valgus group showed a nonsignificant trend below 70% (*p* = 0.098).

**Table 4:** Results of other studies reported in the literature for varus knees.

Authors (year)	Number of knees (male/female)	Methodology	*m* (mm)	*l* (mm)	*d* (mm)	*θ* (°)
Asada et al. (2012) [3]	35 (NR)	CT arthrography	0.39 ± 0.53	1.55 ± 0.26	NR	1.1 ± 0.7 (−0.3, 2.1)
Tashiro et al. (2012) [22]	40 (9/31)	MRI	0.3 ± 0.2/0.4 ± 0.2	1.7 ± 0.6/1.8 ± 0.5	NR	1.7 ± 0.7/1.5 ± 0.7
Fujii et al. (2012) [8]	184 (12/100)	Cartilage excision by scalpel	0.7 ± 0.7 (0.0, 3.3)	2.1 ± 0.7 (0.0, 3.8)	NR	1.7 ± 1.3 (0.0, 4.6)
Hamada et al. (2017) [11]	98 (19/67)	MPR imaging and navigation	—	—	NR	1.7 ± 1.2
Miyasaka et al. (2020) [18]	50 (19/31)	Radiograph	0.6 ± 0.5 (0.0, 2.1)	1.8 ± 0.6 (0.4, 3.0)	46.1 ± 3.3 (39.0, 53.0)	1.5 ± 0.9 (−0.1, 3.9)

*Note*: Data are presented as the mean ± standard deviation and range, as appropriate.

Abbreviations: CT, computed tomography; MPR, multiplanar reformation; MRI, magnetic resonance imaging; NR, not reported.

**Table 5:** Distribution of posterior condylar cartilage thickness strata (<1.5, 1.5–2.5, > 2.5 mm) between female varus (*l*) and valgus (*m*) groups.

Thickness (mm)	Female varus (*l*)	Female valgus (*m*)	*p* value^a^
<1.5	13/31 (41.9%)	6/25 (24.0%)	0.26
1.5–2.5	15/31 (48.4%)	14/25 (56.0%)	0.60
>2.5	3/31 (9.7%)	5/25 (20.0%)	0.45
Total	31 (100%)	25 (100%)	

*Note*: Global *p* value from a 2 × 3 *χ* ^2^ test of independence: *χ* ^2^(2) = 2.50, *p* = 0.29 (Cramér's *V* = 0.21).

^a^ Row‐wise *p* values are two‐sided Fisher's exact tests comparing varus versus valgus within each thickness stratum.

## DISCUSSION

### Key findings

In this female‐only cohort, *C*_diff_ showed an almost perfectly linear association with theoretical FC rotation (slope = 1.31°/mm; *R* ^2^ = 0.99). The slope angle was consistent between valgus and varus knees (1.30 and 1.34°/mm), indicating alignment independence. Rotational deviations up to 4° were observed, underscoring clinical relevance.

### Comparison with prior work

Prior varus knees studies [3, 8, 11, 22] report rotational effects of residual posterior condylar cartilage of approximately 1.1°–1.7°, consistent with the ≈1.5° average (Table 4). In contrast, evidence specific to valgus knees is scarce; to the best of authors' knowledge, only two studies have examined this issue using different methods [16, 20]. A study using MRI [20] found no significant association in valgus knees, and in varus knees, a female‐only effect was detectable but minimal (0.2°). The other study, based on intraoperative gap data without imaging‐based cartilage‐thickness measurements, likewise found no significant influence and included no varus comparators [16]. In addition, Tsukeoka and Tsuneizumi [23] reported that postoperative FC rotation based on posterior condylar referencing is difficult to predict preoperatively, underscoring the limitations of fixed‐thickness assumptions and the need for individualised intraoperative assessment. More recently, Giurazza et al. [9] compellingly demonstrated the feasibility of intraoperative femoral cartilage‐thickness measurement using a photographic reference. The single‐exposure radiographic approach used in this study builds upon and complements that work: whereas photographic benchmarks involve visual identification of the cartilage–bone interface, radiography emphasises the mineralised junction and may help mitigate visual boundary ambiguity.

### Methodological strengths and novelty

Considering limitations of prior reports, radiographic cartilage thickness was estimated with a single‐exposure trigonometric method that leverages differential X‐ray attenuation between subchondral bone and residual cartilage, enabling direct numeric assessment without navigation or MRI. These methodological refinements—together with a balanced varus–valgus cohort—position this study, to the best of authors' knowledge, as the first to provide statistically and clinically significant evidence that residual posterior condylar cartilage directly influences FC rotation in TKA. The findings suggest that cartilage‐remnant asymmetry should inform preoperative risk awareness and intraoperative rotational referencing, rather than prescribing a fixed preoperative rotation, in both mechanically aligned (MA) and kinematically aligned (KA) TKA.

This approach addresses key sources of bias in prior CT/MRI‐based and nonimaging estimates—namely, variability due to imaging resolution, potential deviations from preoperative three‐dimensional plans and the absence of direct measurement of resected osteochondral fragments—while retaining simplicity and high reproducibility as a modification of the Fujii plain‐radiography method [8]. To the best of authors' knowledge, it also extends this correlation to lateral‐compartment OA.

### Clinical implications

Rotational error has well‐documented sequelae: femorotibial rotational mismatch is linked to patellofemoral dysfunction, instability and polyethylene wear [15], while FC malrotation is associated with patellar maltracking [21], residual pain and reduced satisfaction [24]. In this cohort, cartilage‐remnant asymmetry produced deviations up to 4°, underscoring the need for careful intraoperative referencing. While Nam et al. [19] suggested that unworn posterior condylar cartilage is consistently about 2.0 mm regardless of coronal alignment, Klasan et al. [13]—and contemporary intraoperative data such as Campi et al. [5]—demonstrate substantial heterogeneity. This is consistent with a recent MRI‐based systematic review synthesising 27 cohorts (8170 knees), which reported weighted mean distal/posterior femoral cartilage thicknesses of ~1.9–2.5 mm with marked inter‐individual variability, further challenging the fixed 2‐mm assumption [10]. In the female‐only cohort, only about half of knees (≈52%) were within the ‘2 mm range’ (1.5–2.5 mm), and exact binomial tests statistically rejected the prevailing assumption that ≥ 70% of knees fall within this range. These findings provide quantitative evidence that the widely adopted 2‐mm correction is not representative of the actual variability and may therefore introduce systematic bias if applied uniformly. Together with the data from this study, this variability challenges fixed‐thickness corrections and supports cartilage‐adjusted targets when planning FC rotation, and—where available—incorporation into navigation or patient‐specific instrumentation algorithms, recognising that preoperative estimation of cartilage thickness is often difficult.

As shown in Table 5, distributions were broadly similar between deformity types; clinically, in varus knees, overestimation of lateral posterior condylar cartilage may bias the FC toward unintended internal rotation. Current preoperative planning workflows—particularly those based on MA approaches—that rely on plain radiographs or CT scans commonly reference the posterior femoral condyles when setting FC rotation but typically neglect the residual thickness of posterior condylar cartilage. This omission can bias the FC toward unintended internal rotation, particularly when the lateral cartilage is thin—a scenario that may be more frequent in small‐statured populations such as Asian women [18]. Accordingly, quantification of residual posterior condylar cartilage should be incorporated into TKA decision‐making. Although d has been proposed as a knee‐size surrogate, it showed no independent association with theoretical rotation in the female cohort of this study; therefore, it was regarded as a complementary reference rather than a primary determinant. Because preoperative estimation of posterior condylar cartilage thickness is often impractical, a fixed rotation preoperatively based on presumed thickness is not advocated. Instead, awareness of cartilage‐related bias should encourage intraoperative rotational referencing. When thickness can be measured intraoperatively (e.g., by direct measurement of resected fragments or calipered techniques), targeted adjustments may be applied, and otherwise the expected bias should inform postoperative interpretation.

### Limitations and future directions

This study has several limitations. The valgus and varus knee cohorts were recruited at different institutions; however, all surgeries were performed or supervised by the same surgeon under uniform, standardised imaging protocols and measurement techniques, minimising inter‐institutional bias. Furthermore, all participants were of Asian ancestry, potentially limiting the generalisability of the findings from this study to other ethnic groups. Because the underlying mechanism is anatomical, the findings from this study may extend to other small‐stature populations. No male patients were included in the valgus knee cohort, precluding assessment of sex‐related differences in posterior condylar cartilage morphology; thus, applicability to men with lateral‐compartment OA remains uncertain. In addition, the trigonometric radiographic method used in this study, while reproducible, may be sensitive to projection and calibration errors, and cartilage thickness was not directly validated against intraoperative caliper measurements or cross‐sectional imaging in this cohort. Theoretical FC rotation was analysed rather than the actual postoperative component rotation and clinical outcomes were not evaluated. Further prospective, multicenter studies in ethnically diverse cohorts—including men with valgus knees—are warranted to validate cartilage‐adjusted thresholds, correlate them with postoperative rotation and patient‐reported outcomes and establish practical guidelines for both kinematic and mechanical alignment strategies.

## CONCLUSION

In a female‐only cohort of Asian ancestry, residual posterior condylar cartilage asymmetry was almost linearly associated with theoretical FC rotation in TKA. The data provide a cartilage‐adjusted rotational coefficient (≈1.3° of FC rotation per 1‐mm medial–lateral cartilage difference), applicable across valgus and varus knees and particularly pertinent in small‐statured patients. These findings underline the importance of cartilage‐adjusted intraoperative referencing, being relevant not only for KA techniques but also for posterior condylar referencing in MA TKA, while challenging the universality of the 2‐mm correction.

## AUTHOR CONTRIBUTIONS

**Teruyuki Miyasaka**: Conceptualisation. **Teruyuki Miyasaka** and **Tomohiro Kayama**: Methodology. **Teruyuki Miyasaka** and **Taiki Neyatani**: Formal analysis. **All authors**: Investigation. **Teruyuki Miyasaka**: Writing—original draft. **All authors**: Writing—review and editing. **Teruyuki Miyasaka** and **Mitsuru Saito**: Supervision. All authors have read and approved the final manuscript.

## CONFLICT OF INTEREST STATEMENT

Dr. Saito reports grants from Zimmer Biomet Japan, Inc. and grants from Smith & Nephew KK., outside the submitted work. The remaining authors declare no conflict of interest.

## ETHICS STATEMENT

The study protocol was approved by the institutional review board of The Jikei University School of Medicine (Approval No. 33‐464 [11091]). All procedures conformed to the 1964 Helsinki Declaration and its later amendments. Written informed consent was obtained from all individual participants included in this study.

## Supporting information

Supplementary Material

## Data Availability

The data sets generated and analysed during the current study are available from the corresponding author on reasonable request.
