# Supplementary material for: Posterior condylar cartilage thickness determines accuracy of femoral component rotation during total knee arthroplasty in valgus knees: Comparison with varus knees
Source: J Exp Orthop. 2025 Nov 3;12(4):e70486. doi: 10.1002/jeo2.70486 (PMC12581805; doi:10.1002/jeo2.70486)
Supplement: Supplementary file 1 — Supplementary Material [file JEO2-12-e70486-s001.docx]

Supplementary Material for

**Posterior-Condylar Cartilage Thickness Determines Accuracy of Femoral Component Rotation During Total Knee Arthroplasty in Valgus Knees: Comparison With Varus Knees**

**Contents**

Supplementary Tables 1

Supplementary Tables 2

**Supplementary Table 1**. Observer reliability (observers A and B) for posterior condylar measurements

| **Region** | **Variable** | **Intraobserver ICC (A)** | **MAD (A), mm** | **Intraobserver ICC (B)** | **MAD (B), mm** | **Interobserver ICC** | **MAD (AB), mm** |
| --- | --- | --- | --- | --- | --- | --- | --- |
| **Medial** | Total condylar thickness | 0.995 | 0.24 | 0.996 | 0.13 | 0.974 | 0.27 |
|  | Cartilage thickness | 0.996 | 0.12 | 0.997 | 0.07 | 0.993 | 0.12 |
| **Lateral** | Total condylar thickness | 0.993 | 0.16 | 0.997 | 0.13 | 0.984 | 0.19 |
|  | Cartilage thickness | 0.987 | 0.07 | 0.997 | 0.04 | 0.952 | 0.10 |

ICC, intraclass correlation coefficient (2, 1); MAD, mean absolute difference between paired measurements.

**Supplementary Table 2**. Distribution of posterior condylar cartilage thickness strata (<1.5, 1.5–2.5, >2.5 mm) by group

|  | Male varus (l) | Female varus (l) | Female valgus (m) | *P*-value^†^ |
| --- | --- | --- | --- | --- |
| <1.5 mm | 2/19 (10.5%) | 13/31 (41.9%) | 6/25 (24.0%) | 0.048* |
| 1.5-2.5 mm | 17/19 (89.5%) | 15/31 (48.4%) | 14/25 (56.0%) | 0.012* |
| >2.5 mm | 0/19 (0.0%) | 3/31 (9.7%) | 5/25 (20.0%) | 0.10 |
| Total | 19 (100.0%) | 31 (100%) | 25 (100%) |  |

^†^ Row-wise Pearson’s chi-square test of independence (3×2, two-sided) comparing the three groups within each stratum; no multiplicity adjustment (exploratory). *P < 0.05.

Global (3×3) test across all strata and groups: χ²(4) = 11.88, p = 0.018.

Note: Male data are exploratory and reported in the Supplement only; they were not included in the prespecified primary analyses.
